# Supplementary material for: Molecular Encapsulation from the Liquid Phase and Graphene Nanoribbon Growth in Carbon Nanotubes
Source: J Phys Chem Lett. 2022 Oct 12;13(41):9752–8. doi: 10.1021/acs.jpclett.2c02046 (PMC9589896; doi:10.1021/acs.jpclett.2c02046)
Supplement: Supplementary file 1 — jz2c02046_si_001.pdf [file jz2c02046_si_001.pdf]

Supporting Information

Molecular Encapsulation from the Liquid Phase and Graphene Nanoribbon Growth in Carbon Nanotubes

Ana Cadena,[*,†,‡] Bea Botka,[*,†] Áron Pekker,[†] Cla Duri Tschannen,[¶] Chiara Lombardo ,[¶] Lukas Novotny,[¶] Andrei N. Khlobystov,[§] and Katalin Kamarás[†]

†Wigner Research Centre for Physics, Institute for Solid State Physics and Optics, 1525 Budapest, Hungary

‡Department of Chemical and Environmental Process Engineering, Faculty of Chemical Technology and Biotechnology, Budapest University of Technology and Economics, 1111 Budapest, Hungary

¶Photonics Laboratory, ETH Zürich, 8093 Zürich, Switzerland

§Department of Chemistry, University of Nottingham, Nottingham, NG7 2RD, United Kingdom

[*]E-mail: anacristina.cadena@wigner.hu; botka.bea@wigner.hu

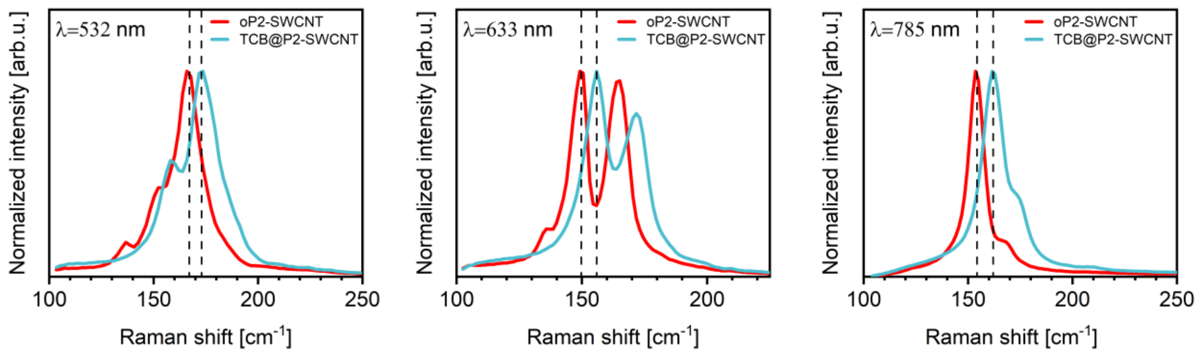

Figure S1. Raman spectra of empty and TCB-filled nanotubes in the RBM region measured with different laser excitation.

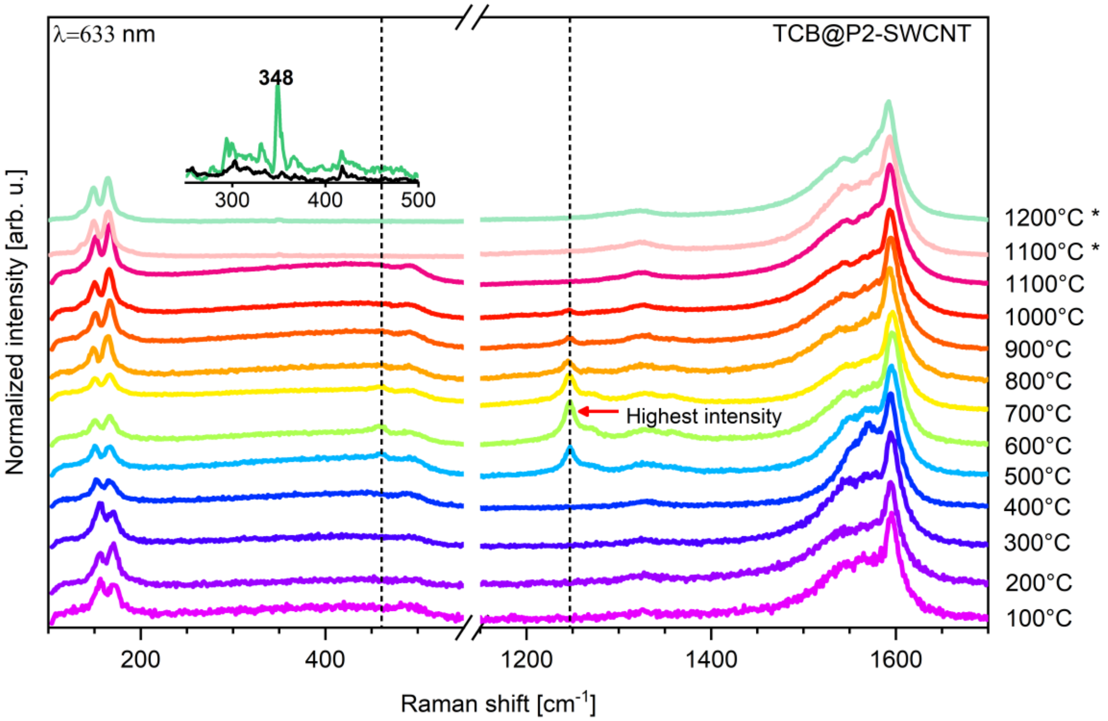

Figure S2. Raman spectra following the ribbon formation, consecutively annealing from 100 °C to 1200 °C in 100 °C increments. The highest intensity peaks of the 6-AGNR, indicated by dashed lines, appeared at 500 °C and 600 °C. The spectra were measured through the quartz tube, except for the temperatures marked with *, where the quartz tube was opened and the spectra were measured straight on the sample. The inset shows the RBM region of the inner tubes of the double-walled nanotubes formed at 1200 °C (green) vs the SWCNT with open caps (black).

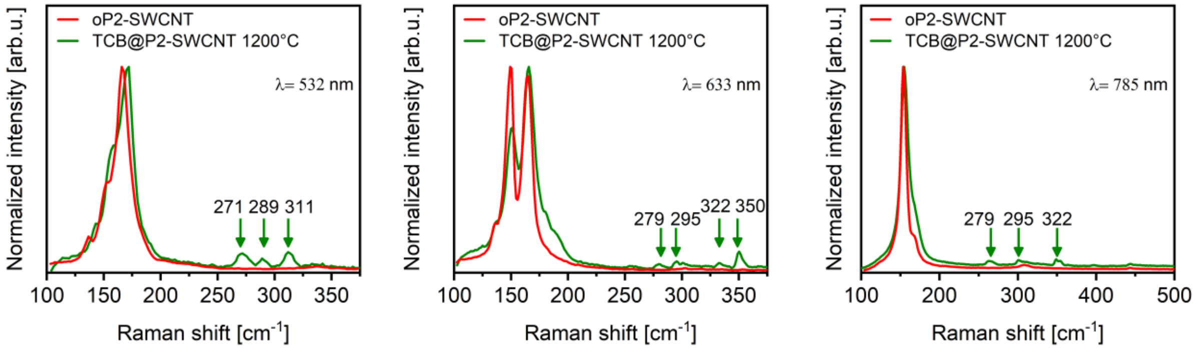

Figure S3. The RBM region of double-walled carbon nanotubes formed at 1200 °C after annealing the TCB@P2 SWCNT 600 °C sample.

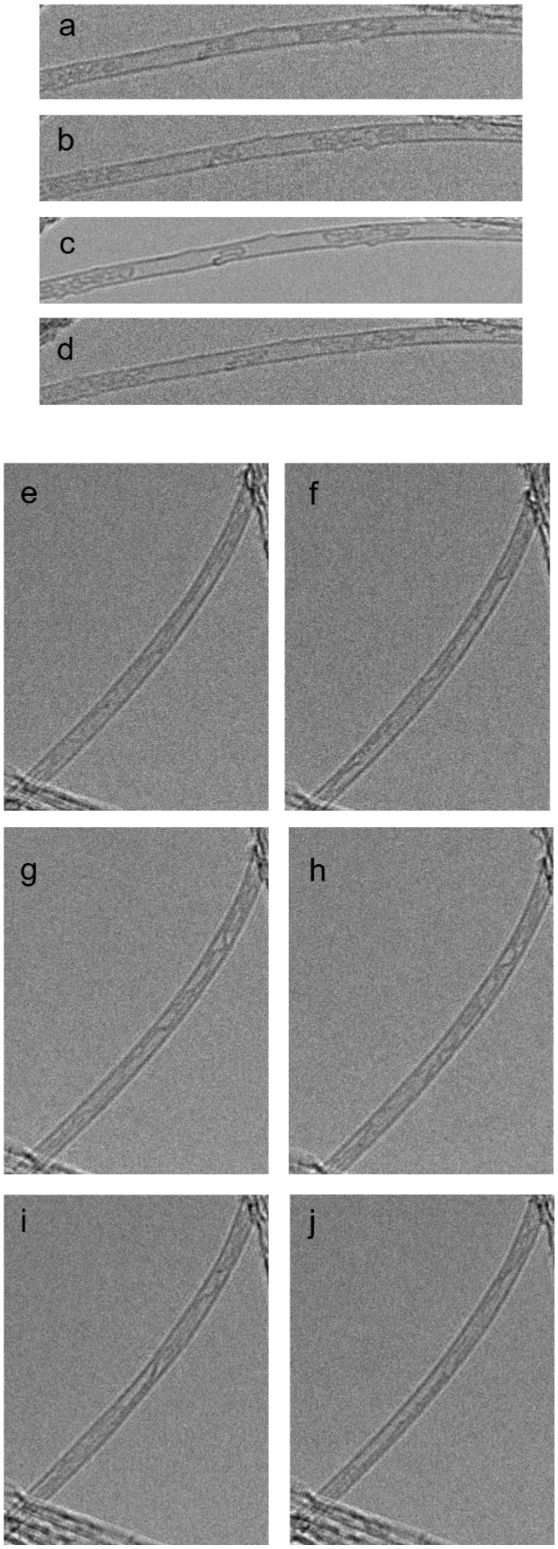

Figure S4. Time series TEM images on nanoribbons formed inside the nanotubes at 500 °C (a,b,c,d) and 600 °C (e,f,g,h,i,j) annealing temperature.

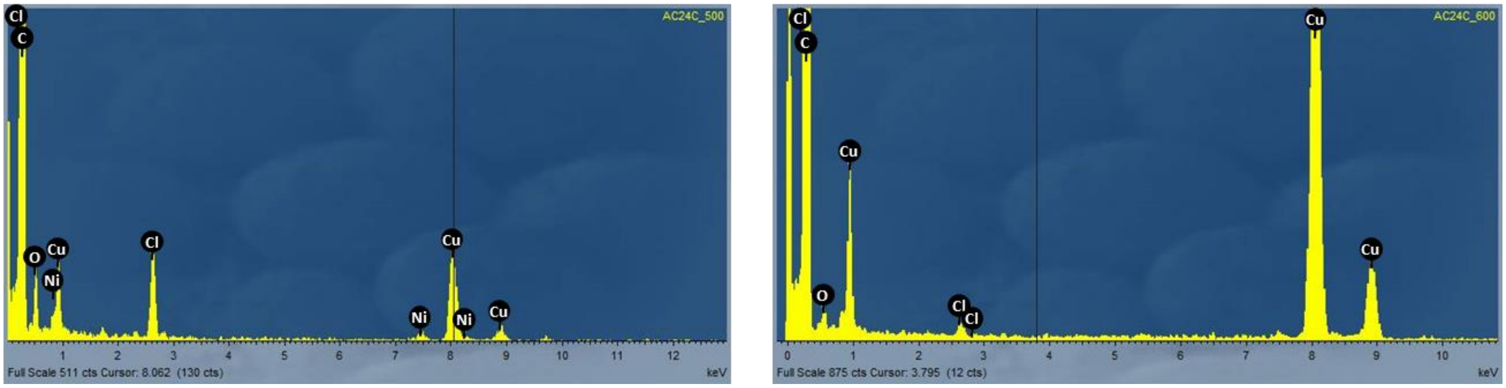

Figure S5. EDS spectra of the ribbons formed at a) 500°C and b) 600° C.

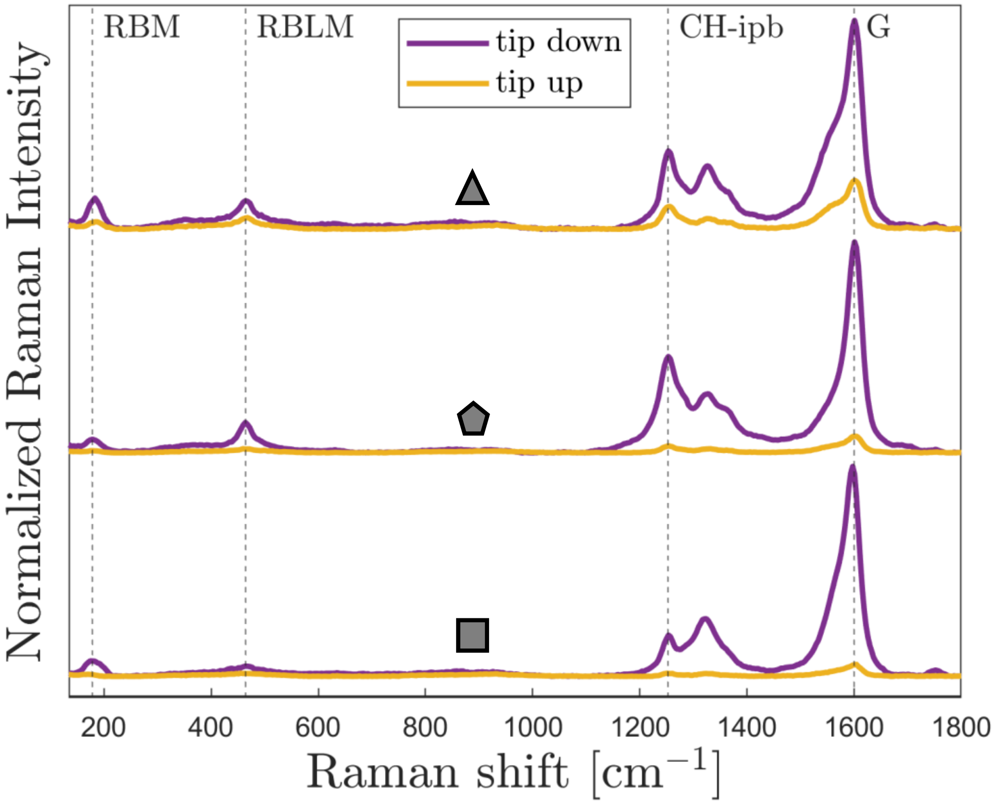

Figure S6. Raman spectra of GNR@P2-SWCNT recorded at the locations marked in Fig. 5(d) with (tip down) and without (tip up) the presence of the TERS probe. All spectra are normalized to the G peak height of the TERS measurements and vertically offset for better visibility.

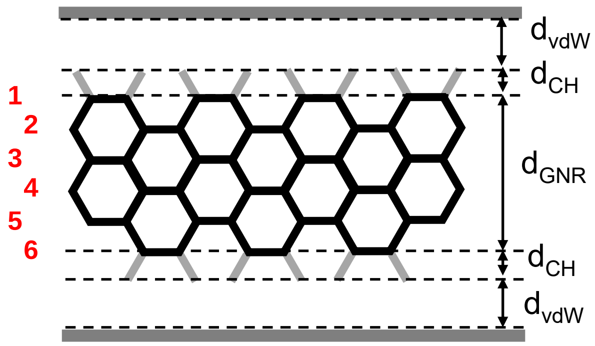

Figure S7. Calculation of the width of armchair (AGNR) nanoribbons. The model is based on simple geometric considerations, using a_{CC}=0.142, a_{CH}=0.11 as carbon-carbon and carbon-hydrogen bond length respectively. The van der Waals distance between the hydrogen and the carbon nonotube wall was calculated based on the van der Waals radii of the atoms (d_{vdW}=0.29 nm).

N	AGNR	ZGNR	AGNR+H	ZGNR+H	AGNR+H+vdW	ZGNR+H+vdW
2	0.123	0.284	0.314	0.504	0.894	1.084
3	0.246	0.497	0.436	0.717	1.016	1.297
4	0.369	0.71	0.559	0.93	1.139	1.51
5	0.492	0.923	0.682	1.143	1.262	1.723
6	0.615	1.136	0.805	1.356	1.385	1.936
7	0.738	1.349	0.928	1.569	1.508	2.149
8	0.861	1.562	1.051	1.782	1.631	2.362

Table S1. Width of the nanoribbons with different N-numbers. Column AGNR and ZGNR contains the width of the bare carbon network. AGNR+H and ZGNR+H includes the terminating hydrogens. The last two columns (AGNR+H+vdW, ZGNR+H+vdW) incorporates the van der Waals distance between the carbon nanotube wall and the terminating hydrogens. Green background indicates the ribbons which can be fitted inside the CNTs considering the threshold for ribbon formation.
