# Supplementary material for: Molecular Encapsulation from the Liquid Phase and Graphene Nanoribbon Growth in Carbon Nanotubes
Source: J Phys Chem Lett. 2022 Oct 12;13(41):9752–8. doi: 10.1021/acs.jpclett.2c02046 (PMC9589896; doi:10.1021/acs.jpclett.2c02046)
Supplement: Supplementary file 2 — jz2c02046_si_002.pdf [file jz2c02046_si_002.pdf]

jz-2022-02046p.R1

Name: Peer Review Information for "Molecular encapsulation from the liquid phase and graphene nanoribbon growth in carbon nanotubes"

First Round of Reviewer Comments

Reviewer: 1

Comments to the Author

The authors show a new method to synthesize graphene nanoribbons inside carbon nanotubes. As was shown previously in several publications, some of which are referenced in this work, annealing organic molecules after encapsulation inside carbon nanotubes results in the transformation of those organic molecules into graphene nanoribbons (see e.g. reference https://www.nature.com/articles/ncomms3548). Showing "another" organic molecule that converts into a graphene nanoribbon might therefore not be particularly appealing on its own. However, the authors are correct that there are issues with the removal of the precursor molecules and subsequent contamination in samples for some of the proposed precursors (not for all!) and that filling with a liquid is much easier than filling from a powder. This thus might provide a good alternative, but only if the overall synthesis yield, length of the ribbons and purity (number of defects, …) of the resulting GNRs are of similar (if not better) quality of those already reported in literature.

In this respect, one could argue that the yield of the synthesis can be quantitatively judged by the relative ratio of the GNR Raman peaks versus the CNT peaks in RRS, certainly when considering the same starting SWCNT batches and the same excitation wavelength. However, when comparing with e.g. the reference: Small Methods 2022, 2200110; the relative intensities are very different, and much lower "yields" are found in this work.

One could also argue that the width of the Raman lines of the GNRs observed in these polydisperse CNT samples, gives some indication of sample purity (number of defects, length of the ribbons, …) Indeed, for less defective GNRs, typically much narrower widths are observed, and if indeed the GNRs have little defects, they should all give the same Raman line, and inhomogeneous broadening should be largely absent. However, when comparing the width of the Raman lines in this work (as extracted manually from the graphs since the data is not provided), with those observed for other literature works, they seem to be much broader than the ~10 cm-1 widths observed in the best references (e.g. https://doi.org/10.1007/s12274-021-3819-8).

I am therefore not convinced that the proposed methodology will give a decent alternative to the procedures already well described in literature. Nevertheless, it does provide interesting data, which might warrant publication, even though I believe that the manuscript requires significant rewriting and contains several open questions that need to be resolved first. Below I give a more detailed list of

remaining questions, that should be addressed before publication. These detailed comments are given in no particular order of importance.

- In their abstract, the authors claim to ONLY synthesize 6-AGNR nanoribbons without any byproducts. I cannot agree that this is proven by their data. They can assume there are no by products because they start from the solvent, but how can they prove it? There might be many by products that can be formed. Moreover, they observe RBLMs at different frequencies (446 and 457) which shows these are different GNRs. Moreover, they ONLY use 3 excitation wavelengths, two of which are chosen in resonance with the 6AGNR transitions according to reference 8. Therefore it feels like a circular reasoning – they only verify the growth of 6GNRs, and then use that to claim they only grow 6GNRs. These statements should be carefully readdressed in the text and abstract.

- Overall, the manuscript is poorly written, often very short, incomplete sentences are used. I advice the authors to let the manuscript be read by a native English speaker.

- Very often, I also miss a reference to the literature for specific statements. For example, in the introduction : "This not only leads to lower encapsulation ratios compared to direct encapsulation methods, but can also alter the reaction pathway of the precursor inside the cavity."

- When the authors refer to the RBM shifts of filled tubes, they state that "the RBM of the filled tubes was always blueshifted by up to 2 cm−1 compared to the empty ones." This is not correct. In fact, it was observed that the shift is very different for different SWCNT diameters, starting from approximately 1.4-3 cm-1 for small diameter SWCNTs upon water filling (https://doi.org/10.1103/PHYSREVLETT.104.207401), but easily amounting to larger shifts (5cm-1) for SWCNTs filled with for example linear alkanes (see e.g. https://doi.org/10.1039/C6NH00062B) or other solvent molecules (https://doi.org/10.1021/acsnano.0c08352). Hence the "up to 2cm-1" statement should be corrected for.

- The authors state that a shift of 7 cm-1 was observed for TCB filling, but surprisingly after conversion to GNRs this shift is lost again. Note that they measure in bundles, and that the CNTs can become more bundled by the immersion in TCB and subsequent filtration, which can explain the large shift.

It is intriguing in view of the RBM shift originating from a steric hindrance of the inwards vibration of the CNTs, one would intuitively expect for a GNR that is synthesized to fit exactly inside the CNTs to lead to a stronger RBM shift than the much smaller solvent molecules that could perhaps more easily adopt. The authors do not comment on the fact that the shift is lost when converting to GNRs. This seems to indicate that the TCB is removed from the CNTs upon annealing, and that only a minor fraction of the CNTs is filled with GNRs (as also shown in the TEM observations). The authors should comment more on this in their manuscript.

This then calls the question: How can the authors prevent the small, volatile TCB molecules to not exit the CNTs while annealing to high temperature and instead form a nanoribbon. Perhaps that is the reason of the low yields?

- Figure 1 i: it is important to include at this point the exact procedure used (is the sample each time consecutively annealed further (and for how long?) or are those spectra from each time a different sample, annealed for the same time at a different temperature). The consecutive annealing might give

very different results than the direct annealing, so the authors should comment on this in the main text (I realize it is kind of explained in the methods section, but it is needed in the main text to highlight this).

- Given my comment above regarding the line widths, could the authors give a statement on the line widths of the RBLMs and CH-ipb and DLM modes? In particular at 532nm in figure 2a, a very broad RBLM is observed, which is indicative of very defective GNRs synthesized inside. This should be commented on and compared with literature data

- The tip-enhanced Raman spectra are a nice addition that has never been shown to my knowledge for encapsulated GNRs, but unfortunately occurred on bundles. Why was a bundle chosen? Is it needed to get sufficient signal intensity? The authors only show one example, is this a representative figure for many observations or did they only observe one structure? From this it seems there is a high degree of filling along the length of the CNT (what is the spatial resolution?), but how does that correlate with the relatively low intensities with respect to the RRS of the CNTs in the bulk samples?

Reviewer: 2

Comments to the Author

The manuscript by Cadena et al. reports a study on the growth of graphene nanoribbons (GNRs) using single wall carbon nanotubes (CNTs) as a template. The authors utilize 1,2,4-trichlorobenzene as the source for the GNR growth. The developed process is novel, and it has several advantages over previously reported methods: it is simple, does not utilize solvent and the small organic molecule-precursor can be easily removed by evaporation leaving clean carbon nanotube-GNR hybrid material. GNRs are characterized with spectroscopic and microscopic techniques. The study is original, and it contributes to the field of carbon nanomaterials and in particular graphene nanoribbons, a novel electronic material that attracts significant research interest. I recommend publication after major revision to address the following points:

1. All abbreviation should be spelled out the first time they appear in the manuscript. For example, I assume that RBLM is radial breathing-like mode; AGNR is armchair GNR, etc.

2. A brief description of nanoribbons 6-AGNR and 7-AGNR is recommended. I was not familiar with the terminology and whereas it is common for a reviewer to do additional literature search to evaluate the manuscript, it is likely that the regular reader would disregard the article or miss key aspects of the study.

3. What is the physical origin of RBLM of GNRs?

4. The authors have the capability to study individual carbon nanotubes; Figure 5 – tip-enhanced Raman spectroscopy. Was there a correlation between the SWNT diameter (diameter distribution 1.2-1.6 nm) and the growth of GNRs?

5. The presence of Cl in the formed GNRs may be important for their electronic properties as it may affect charge distribution and it may lead to doping of the GNRs. Can XPS be used to determine Cl in the material (if not at this stage, presumably in future studies)?

6. Any comment about the length of the formed GNRs? If precise length estimation is not possible, the authors can at least discuss approximate length or length range.

Reviewer: 3

Comments to the Author

The fabrication of graphene nanoribbons (GNRs) has received continued attentions for their potential applications in the nanoelectronics and their growth in carbon nanotubes (CNTs) is one of the typical methods for selectively making sub-nanometer GNRs. In this work, the authors report selective synthesis of 6-armchair GNRs (6-AGNRs) in CNTs using 1,2,4-trichlorobenzene (TCB) as the precursor. Since TCB is a liquid at room temperature, its use as the precursor facilitates its filling into the CNTs and the subsequent removal of the excess TCB. The characterization of the resulting GNRs was performed by Raman, TEM, and tip-enhanced Raman analyses, and high-quality of the obtained 6-AGNR was especially corroborated by Raman spectra. This work demonstrates the simplification of the growth of GNRs in CNTs using low-cost and readily available TCB as the precursor, while achieving high selectivity and quality in the fabrication of 6-AGNR. Therefore, this reviewer considers that this work can be qualified for the publication in the Journal of the Physical Chemistry Letters after a few minor revisions as listed below:

1. In the abstract, the authors claim that the “procedure results in 6-armchair graphene nanoribbon without byproducts.” However, in the Raman spectra, for example in Figure 6d, an additional RBLM mode is visible at ~300-350 nm as a broad feature, possibly from broader GNRs with less defined structures. The RBLM peak assigned to the 6-AGNR is seemingly not very sharp, although not explicitly discussed, and there is visibly a shoulder, which might be due to some byproducts with defective edge structures. The above claim should thus be appropriately revised or more clarifications should be added to support it.

2. Related to the previous point, the RBLM can be a good indicator of the width of the GNRs, but not unambiguously prove the precise edge structures. To this end, the other observed Raman peaks should also be discussed in comparison to theoretical values, to evidence the armchair edge structure without significant defects. There might also be peaks related to the remaining chloro groups.

3. In the introduction, the authors wrote “Oriented chemical reactions on surfaces, starting from small planar molecules, were the first attempts at bottom-up techniques.” However, there were older attempts by the solution chemistry. For example, Macromolecules 2003, 36, 7082; J. Am. Chem. Soc. 2008, 130, 4216.

Author's Response to Peer Review Comments:

Reply to Reviewers' comments

Reviewer: 1

We thank the Reviewer for the detailed and expert comments that helped us to improve the manuscript. Below, we give our responses to the Reviewer's comments.

The authors show a new method to synthesize graphene nanoribbons inside carbon nanotubes. As was shown previously in several publications, some of which are referenced in this work, annealing organic molecules after encapsulation inside carbon nanotubes results in the transformation of those organic molecules into graphene nanoribbons (see e.g. reference https://www.nature.com/articles/ncomms3548). Showing "another" organic molecule that converts into a graphene nanoribbon might therefore not be particularly appealing on its own. However, the authors are correct that there are issues with the removal of the precursor molecules and subsequent contamination in samples for some of the proposed precursors (not for all!) and that filling with a liquid is much easier than filling from a powder. This thus might provide a good alternative, but only if the overall synthesis yield, length of the ribbons and purity (number of defects, ...) of the resulting GNRs are of similar (if not better) quality of those already reported in literature.

Nanoribbons are a very promising family of materials, and at this stage of our knowledge, the state of the art is still very much the building of a library of various ribbons by as many methods as possible. We show here a facile and fast method to prepare encapsulated nanoribbons. Not all organic molecules transform into ribbons, and presently, the selection of possible precursors and pathways is mainly done by trial and error. (We have summarized the linear ribbons grown inside nanotubes in our review paper (https://academic.oup.com/ooms/article/1/1/itab009/6296610) and for the twisted ribbons mentioned in the paper by Lim et al. there is only indirect evidence.) Our goal was to contribute to such efforts and show a possible direction where to continue. All other data in the literature are far from perfection in terms of the parameters the Reviewer cites, but they all represent important contributions to widening the circle of possible nanoribbons.

In this respect, one could argue that the yield of the synthesis can be quantitatively judged by the relative ratio of the GNR Raman peaks versus the CNT peaks in RRS, certainly when considering the same starting SWCNT batches and the same excitation wavelength. However, when comparing with e.g. the reference: Small Methods 2022, 2200110; the relative intensities are very different, and much lower "yields" are found in this work.

Relative Raman intensities depend on resonance conditions. The ratio of GNR peaks to CNT peaks is determined by the respective species that are in resonance with the exciting laser, and therefore is determined by the concentration of the given CNT in the sample, i.e. the chirality distribution of the starting nanotube ensemble. As the Reviewer notes, comparison can only be made considering the same starting SWCNT batches, and ours was quite different from the one used in the cited paper. We want to emphasize again, though, that we wanted to present a proof of concept, and believe that improving the yield can be done at a later stage when the community, by joint efforts, has determined the starting materials and methods optimal for special applications.

One could also argue that the width of the Raman lines of the GNRs observed in these polydisperse CNT samples, gives some indication of sample purity (number of defects, length of the ribbons, ...) Indeed, for less defective GNRs, typically much narrower widths are observed, and if indeed the GNRs have little defects, they should all give the same Raman line, and inhomogeneous broadening should be largely absent. However, when comparing the width of the Raman lines in this work (as extracted manually from the graphs since the data is not provided), with those observed for other literature works, they seem to be much broader than the 10 cm-1 widths observed in the best references (e.g. https://doi.org/10.1007/s12274-021-3819-8).

I am therefore not convinced that the proposed methodology will give a decent alternative to the procedures already well described in literature.

We do not think of our approach as an alternative, but as an extension to already existing methods. The literature contains relatively few examples of encapsulated nanoribbons, considering the theoretical possibilities. Publications on the subject differ in the host nanotubes and the encapsulated precursors, and produce specific nanoribbons depending on the parameters of both the materials and the procedures. We present here a simple, clean process that applies an easily available precursor that can possibly adapt to the diameter of the host.

Nevertheless, it does provide interesting data, which might warrant publication, even though I believe that the manuscript requires significant rewriting and contains several open questions that need to be resolved first. Below I give a more detailed list of remaining questions, that should be addressed before publication. These detailed comments are given in no particular order of importance.
- In their abstract, the authors claim to ONLY synthesize 6-AGNR nanoribbons without any byproducts. I cannot agree that this is proven by their data. They can assume there are no by products because they start from the solvent, but how can they prove it? There might be many by products that can be formed. Moreover, they observe RBLMs at different frequencies (446 and 457) which shows these are different GNRs. Moreover, they ONLY use 3 excitation wavelengths, two of which are chosen in resonance with the 6AGNR transitions according to reference 8. Therefore it feels like a circular reasoning – they only verify the growth of 6GNRs, and then use that to claim they only grow 6GNRs. These statements should be carefully readdressed in the text and abstract.

This is a very important comment and we thank the Reviewer to have brought it to our attention before publication. Of course, claiming that only 6-AGNRs grow is a mistake based on the resonance conditions used. We have included an estimation of possible ribbons based on their size relative to the diameter distribution in the starting P2 nanotube ensemble. We arrive at the conclusion that 6-AGNRs can be positively identified, and mention the other possibilities as well.

About the by-products, TEM images (a representative amount, not just one scan) show the outside of the tubes to be extremely clean, therefore we assume that annealing produces ribbons only, although the Reviewer is perfectly right that these are not exclusively of one type.

Overall, the manuscript is poorly written, often very short, incomplete sentences are used. I advice the authors to let the manuscript be read by a native English speaker.

We gave the manuscript a thorough language revision.

Very often, I also miss a reference to the literature for specific statements. For example, in the introduction : "This not only leads to lower encapsulation ratios compared to direct encapsulation methods, but can also alter the reaction pathway of the precursor inside the cavity."

We did not mean a specific example, as in our opinion there are many more possibilities in this direction as have been already tried, but inserted a reference to chemical reactions of encapsulated species in general (https://doi.org/10.1039/C6CS00090H). In other parts of the manuscript, we inserted references to support our statements.

When the authors refer to the RBM shifts of filled tubes, they state that "the RBM of the filled tubes was always blueshifted by up to 2 cm-1 compared to the empty ones." This is not correct. In fact, it was observed that the shift is very different for different SWCNT diameters, starting from approximately 1.4-3 cm-1 for small diameter SWCNTs upon water filling (https://doi.org/10.1103/PHYSREVLETT.104.207401), but easily amounting to larger shifts (5cm-1) for SWCNTs filled with for example linear alkanes (see e.g. https://doi.org/10.1039/C6NH00062B) or other solvent molecules (https://doi.org/10.1021/acsnano.0c08352). Hence the "up to 2cm-1" statement should be corrected for.

We thank the Reviewer to have raised our attention to this oversight. The data we referred to were compared to water-filled nanotubes, hence the shift was smaller. The Reviewer is right: compared to empty nanotubes, the shift can easily be much larger. We corrected the text accordingly.

The authors state that a shift of 7 cm-1 was observed for TCB filling, but surprisingly after conversion to GNRs this shift is lost again. Note that they measure in bundles, and that the CNTs can become more bundled by the immersion in TCB and subsequent filtration, which can explain the large shift. It is intriguing in view of the RBM shift originating from a steric hindrance of the inwards vibration of the CNTs, one would intuitively expect for a GNR that is synthesized to fit exactly inside the CNTs to lead to a stronger RBM shift than the much smaller solvent molecules that could perhaps more easily adopt. The authors do not comment on the fact that the shift is lost when converting to GNRs. This seems to indicate that the TCB is removed from the CNTs upon annealing, and that only a minor fraction of the CNTs is filled with GNRs (as also shown in the TEM observations). The authors should comment more on this in their manuscript. This then calls the question: How can the authors prevent the small, volatile TCB molecules to not exit the CNTs while annealing to high temperature and instead form a nanoribbon. Perhaps that is the reason of the low yields?

We agree that the shift originates mostly from strain caused by the encapsulated molecules, though other aspects can be important as well, as discussed in https://doi.org/10.1103/PHYSREVLETT.104.2[illegible] However, we note that even this strain is a though question to address. As to the size of TCB, it is $\sim$ 0.49 and 0.64 nm along the shortest/longest axis, that is not insignificant compared to the ribbon width. Taking into account the van der Waals distance and the intermolecular forces in the liquid, assemblies of TCB molecules can fit tightly into the nanotubes used in this work. Furthermore, as we do not know the exact distribution and geometry of possible ribbons, there is no saying exactly how much strain they will exert on the tube wall. To fully map all possibilities, the whole ensemble of nanotubes (preferably in a DOC solution) should be measured, using a broad range of excitation wavelengths, which is beyond the scope of the

current communication.

On the other hand, the Reviewer is right stating that TCB molecules can exit the nanotubes instead of forming nanoribbons, if the nanotubes are annealed in vacuum at low temperature. In the sample that was prepared using the consecutive annealing sequence, which we started at 100 °C, clearly a lower yield of ribbon formation was observed. Additionally, annealing in dynamic vacuum resulted in a very low yield, which we observed in the case of some other precursors as well. Hence, we repeated the experiment by directly placing the quartz tube into a preheated furnace that was set to the previously identified ideal temperature for ribbon formation. We believe that if the TCB@SWCNT is rapidly heated to the GNR formation temperature, the reaction between the TCB molecules happens before a significant amount of molecules would be able to exit the nanotubes. As it is potentially more difficult for the reaction products to leave the nanotubes, even smaller ribbon fragments can act as plugs to prevent the unreacted TCB molecules from leaving.

A series of TEM images shows that nanotubes are well filled, therefore the lack of RBM shift is not due to a low filling ratio. For the demonstration of shorter vs. longer ribbons we have intentionally chosen images (Fig. 4 a,b), where empty tube sections are present, and the separate short ribbons are well distinguishable.

Figure 1 i: it is important to include at this point the exact procedure used (is the sample each time consecutively annealed further (and for how long?) or are those spectra from each time a different sample, annealed for the same time at a different temperature). The consecutive annealing might give very different results than the direct annealing, so the authors should comment on this in the main text (I realize it is kind of explained in the methods section, but it is needed in the main text to highlight this).

We included a more detailed explanation of the procedure in the text.

Given my comment above regarding the line widths, could the authors give a statement on the line widths of the RBLMs and CH-ipb and DLM modes? In particular at 532nm in figure 2a, a very broad RBLM is observed, which is indicative of very defective GNRs synthesized inside. This should be commented on and compared with literature data

As we discuss in the text, the 6-AGNRs are not expected to be in resonance with the 532 nm excitation used to record spectra in Figure 2a. The ribbons that are in resonance are probably shorter fragments, hence their intensity is much higher when annealed at 500 °C. We expect that with this excitation wavelength, the species that produce the 446 cm^{-1} band are defected ribbon fragments that have not fully transformed to 6-AGNRs. To assess the quality of 6-AGNRs it is more appropriate to analyze spectra in Figure 2d, recorded with 633 nm excitation.

The tip-enhanced Raman spectra are a nice addition that has never been shown to my knowledge for encapsulated GNRs, but unfortunately occurred on bundles. Why was a bundle chosen? Is it needed to get sufficient signal intensity? The authors only show one example, is this a representative figure for many observations or did they only observe one structure? From this it seems there is a high degree of filling along the length of the CNT (what is the spatial resolution?), but how does that correlate with the relatively low intensities with respect to the RRS of the CNTs in the bulk samples?

We thank the Reviewer for appreciating our TERS measurements. The images are indeed representative for multiple investigated locations. The reason that the image shows a bundle is not because of the signal intensity, but simply because the dispersion procedure has not yet been optimized to yield individual tubes. The improvement of this process will allow for detailed TERS studies of single GNR-filled tubes in future studies.
The spatial resolution of the presented TERS measurements is 34 nm, as now indicated in Figure 5(d). Naturally, we cannot make a statement about the continuity of the ribbons below this limit. However, we can conclude that nanoribbon formation occurs throughout the nanotubes and not only in spatially restricted regions.
Regarding the signal intensities, in far-field measurements of bulk samples, the intensity of the GNR spectral signatures relative to those of the CNTs is affected by the presence of empty CNTs. The overall yield, as characterized by the relative GNR Raman signal strength, can potentially be improved by optimizing the diameter distribution of the CNT sample.

Reviewer: 2

We thank the reviewer for the valuable comments on our manuscript. Below, we give our responses to the Reviewer's comments.

The manuscript by Cadena et al. reports a study on the growth of graphene nanoribbons (GNRs) using single wall carbon nanotubes (CNTs) as a template. The authors utilize 1,2,4-trichlorobenzene as the source for the GNR growth. The developed process is novel, and it has several advantages over previously reported methods: it is simple, does not utilize solvent and the small organic molecule-precursor can be easily removed by evaporation leaving clean carbon nanotube-GNR hybrid material. GNRs are characterized with spectroscopic and microscopic techniques. The study is original, and it contributes to the field of carbon nanomaterials and in particular graphene nanoribbons, a novel electronic material that attracts significant research interest. I recommend publication after major revision to address the following points:

1. All abbreviation should be spelled out the first time they appear in the manuscript. For example, I assume that RBLM is radial breathing-like mode; AGNR is armchair GNR, etc.

We added a summary of the Raman modes of both nanotubes and nanoribbons, where we explain the RBM and RBLM vibrations.

2. A brief description of nanoribbons 6-AGNR and 7-AGNR is recommended. I was not familiar with the terminology and whereas it is common for a reviewer to do additional literature search to evaluate the manuscript, it is likely that the regular reader would disregard the article or miss key aspects of the study.

We expanded in the introduction the description of the GNR conformations and added an estimation of the possible nanoribbons conforming to the nanotube batch used. (Supporting Information, Figure S7 and Table S1).

3. What is the physical origin of RBLM of GNRs?

The physical origin of the RBLM has been described (see answer to question 1).

4. The authors have the capability to study individual carbon nanotubes; Figure 5 – tip-enhanced Raman spectroscopy. Was there a correlation between the SWNT diameter (diameter distribution 1.2-1.6 nm) and the growth of GNRs?

From geometrical considerations and Raman intensities with different exciting laser lines, we gave an estimate on possible GNRs that grow inside the nanotubes (Supporting Information, Table S1). Among these, 6-AGNR was positively identified.

5. The presence of Cl in the formed GNRs may be important for their electronic properties as it may affect charge distribution and it may lead to doping of the GNRs. Can XPS be used to determine Cl in the material (if not at this stage, presumably in future studies)?

EDS was used to determine Cl in the 500 and 600 °C annealed sample. Unfortunately, neither C-Cl vibrations in the Raman spectra (expected 200 cm^{-1}) nor images in the TEM picture could be unambigously identified. We added a section to the text concerning this topic.

6. Any comment about the length of the formed GNRs? If precise length estimation is not possible, the authors can at least discuss approximate length or length range.

We thank the Reviewer for mentioning this important point that we omitted from the discussion. Based on the HR-TEM images, we estimate a mean length of 20 nm for the TCB@SWNT500 and ¿ 100 nm for the TCB@SWNT600 sample.

Reviewer: 3

We thank the Reviewer for the valuable comments that helped improve our manuscript. Below, we give our responses to the Reviewer's remarks:

The fabrication of graphene nanoribbons (GNRs) has received continued attentions for their potential applications in the nanoelectronics and their growth in carbon nanotubes (CNTs) is one of the typical methods for selectively making sub-nanometer GNRs. In this work, the authors report selective synthesis of 6-armchair GNRs (6-AGNRs) in CNTs using 1,2,4-trichlorobenzene (TCB) as the precursor. Since TCB is a liquid at room temperature, its use as the precursor facilitates its filling into the CNTs and the subsequent removal of the excess TCB. The characterization of the resulting GNRs was performed by Raman, TEM, and tip-enhanced Raman analyses, and high-quality of the obtained 6-AGNR was especially corroborated by Raman spectra. This work demonstrates the simplification of the growth of GNRs in CNTs using low-cost and readily available TCB as the precursor, while achieving high selectivity and quality in the fabrication of 6-AGNR. Therefore, this reviewer considers that this work can be qualified for the publication in the Journal of the Physical Chemistry Letters after a few minor revisions as listed below: 1. In the abstract, the authors claim that the "procedure results in 6-armchair graphene nanoribbon without byproducts." However, in the Raman spectra, for example in Figure 6d, an additional RBLM mode is visible at 300-350 nm as a broad feature, possibly from broader GNRs with less defined structures. The RBLM peak assigned to the 6-AGNR is seemingly not very sharp, although not explicitly discussed, and there is visibly a shoulder, which might be due to some byproducts with defective edge structures. The above claim should thus be appropriately revised or more clarifications should be added to support it.

We changed the title and refined the reference to 6-AGNR, now saying that this is the type

of ribbons we could positively identify. We added a section of possible other products and why we cannot unambigously prove their presence, but they should not be excluded.

2. Related to the previous point, the RBLM can be a good indicator of the width of the GNRs, but not unambiguously prove the precise edge structures. To this end, the other observed Raman peaks should also be discussed in comparison to theoretical values, to evidence the armchair edge structure without significant defects. There might also be peaks related to the remaining chloro groups.

We added an estimation of possible armchair ribbons, based on geometrical considerations and Raman resonances (Supporting Information, Table S1). For precise determination of the edge structure, more nanoscale measurements (TEM, TERS on individual nanotubes) should be performed, and we plan such measurements in the near future.

3. In the introduction, the authors wrote "Oriented chemical reactions on surfaces, starting from small planar molecules, were the first attempts at bottom-up techniques." However, there were older attempts by the solution chemistry. For example, Macromolecules 2003, 36, 7082; J. Am. Chem. Soc. 2008, 130, 4216.

We thank the Reviewer for directing our attention to these papers that we now cite in the introduction.
